# Primary kidney squamous cell carcinoma, misdiagnosed as xanthogranulomatous pyelonephritis: A case report

**DOI:** 10.1016/j.eucr.2026.103608

**Published:** 2026-09-09

**Authors:** Dorsa Ghasemi, Milad Benam, Marjan Akhavan, Seyed Arman Mousavi, Arash Sharghi Goorabi, Soha Ghasemi

**Affiliations:** a Dr. Tahririan Pathology Lab, Hormozgan University of Medical Sciences, Bastak, Iran; b Farabi Hospital of Bastak, Hormozgan University of Medical Sciences, Iran

**Keywords:** Renal squamous cell carcinoma, Chronic pyelonephritis, Nephrolithiasis, Xanthogranulomatous pyelonephritis

## Abstract

Squamous cell carcinoma (SCC) of the kidney is a rare malignancy, typically arising in the renal pelvis in association with chronic irritation and infection. Persistent inflammation may lead to squamous metaplasia and progression to carcinoma. Preoperative diagnosis is challenging due to nonspecific clinical and radiologic findings, often mimicking inflammatory conditions. We report a case of renal SCC in a nonfunctional kidney with long-standing pyelonephritis and nephrolithiasis, initially suspected as xanthogranulomatous pyelonephritis. The diagnosis was confirmed histopathologically. This case emphasizes the importance of considering malignancy in chronically diseased kidneys and the critical role of histopathologic evaluation.

## 1 Introduction

Squamous cell carcinoma (SCC) of the kidney is an exceptionally rare malignancy, accounting for less than 1% of all renal cancers.^1^ It frequently involves the renal pelvis in the setting of chronic irritation of the urothelium. Reported predisposing factors include chronic nephrolithiasis, recurrent infections, prior radiotherapy, exposure to endogenous and exogenous carcinogens, and vitamin A deficiency.^1^^,^^2^ Owing to its nonspecific clinical and radiologic features, renal SCC is often diagnosed at an advanced stage, resulting in an overall poor prognosis.^1^^,^^2^

In this case report, we describe a rare case of renal SCC in a 55-year-old man who presented with flank pain and prolonged fever and was treated with radical nephrectomy, emphasizing the clinicopathologic features and diagnostic challenges of this uncommon entity.

## 2 Case report

A 55-year-old man presented to the clinic with a one-week history of intermittent, localized, dull, left flank pain, associated with nausea, vomiting, fever, and rigors. He denied any macroscopic hematuria or other urinary symptoms. On physical examination, he revealed left costovertebral angle (CVA) tenderness without abdominal distension. Despite a fatty abdomen, a mass-like lesion was palpated on the left side. Due to initial weight limit restrictions at local outpatient imaging facilities (initial BMI 40 kg/m^2^), prior cross-sectional imaging could not be obtained. Abdominal CT evaluation was successfully performed preoperatively following a substantial unintentional weight loss of 15–20 kg.

Lab investigations revealed normal electrolytes, but an elevated erythrocyte sedimentation rate (ESR) of 65 mm/hr. (reference: 0-20 mm/hr.) and a markedly increased C-reactive protein (CRP) level of 88 mg/L (reference <10 mg/L). The white blood cell (WBC) count was elevated at 24.1 × 10^3^/μL (reference: 4.5–10 × 10^3^/μL). Serum creatinine was within the normal range at 0.9 mg/dL (reference: 0.7-1.2 mg dL), whereas hemoglobin was reduced to 8.8 g/dL.

(reference: 13/5-16/5 mg/dL). Urinalysis showed 2+ leukocytes, 1+ protein, microscopic hematuria, and positive leukocyte esterase. The estimated glomerular filtration rate (eGFR) was 62 mL/min/1.73 m^2^ (reference: >60 mL/min/1.73m**^2^**)**.**

**Fig. 1:**
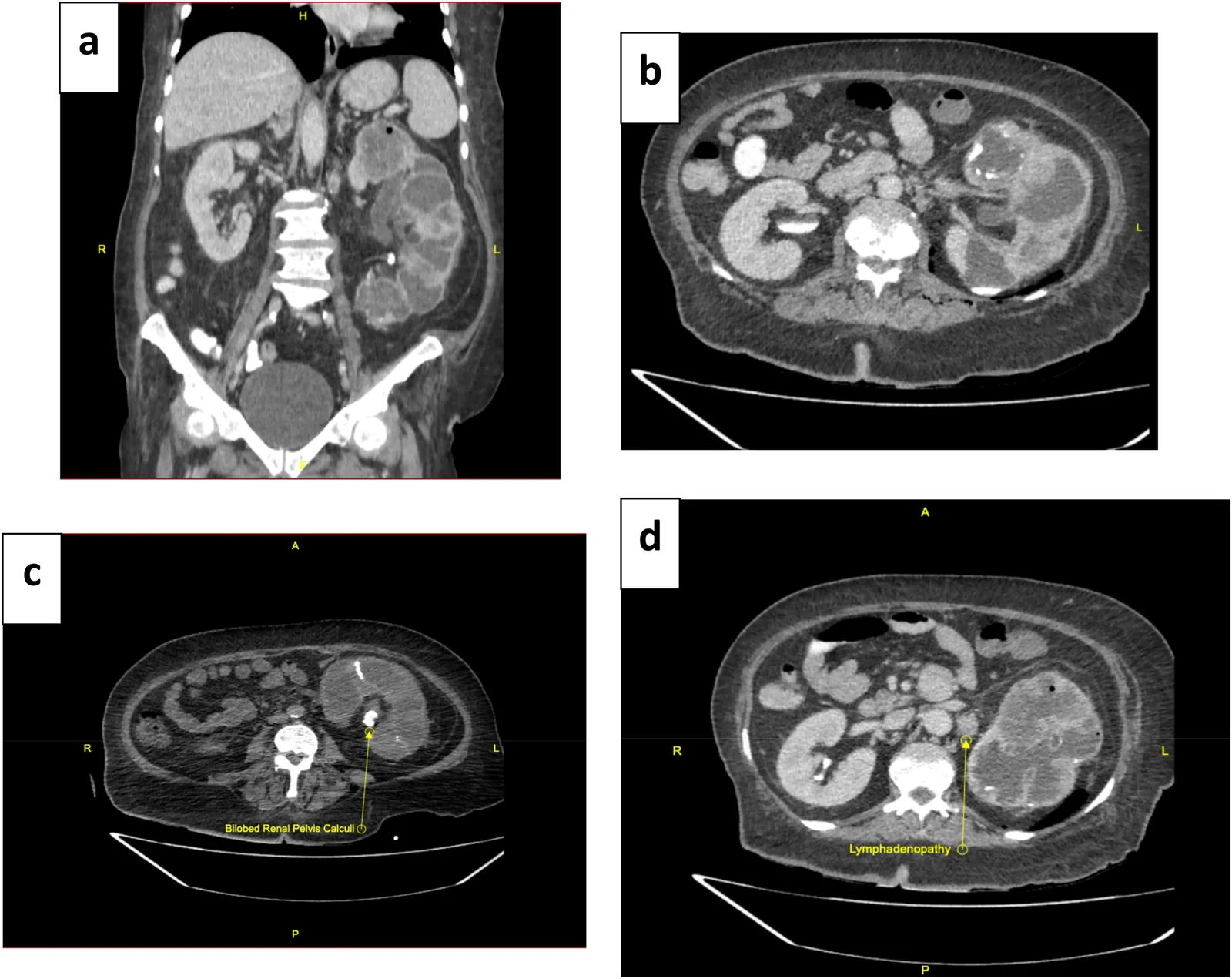
a. Coronal section of the abdomen shows a large left kidney with internal cystic components; b) Axial section of the mid abdomen shows multiple milk of calcium deposits in the left renal calyces; c) Bilobed obstructive renal pelvis stone; d) regional lymphadenopathy.

The patient subsequently underwent an abdominopelvic CT scan. Imaging revealed marked enlargement of the left kidney with severe, prolonged hydronephrosis caused by an obstructing bilobed calculus within the renal pelvis. The renal cortex was diffusely thinned, and the calyces contained multiple deposits of milk of calcium. Additionally, several areas of renal soft-tissue thickening with heterogeneous enhancement were identified within the renal parenchyma. On delayed images, the left kidney showed no normal contrast excretion, consistent with a nonfunctional kidney. Multiple enlarged lymph nodes were also noted in the left para-aortic region of the retroperitoneum (Fig. 1).

**Fig. 2:**
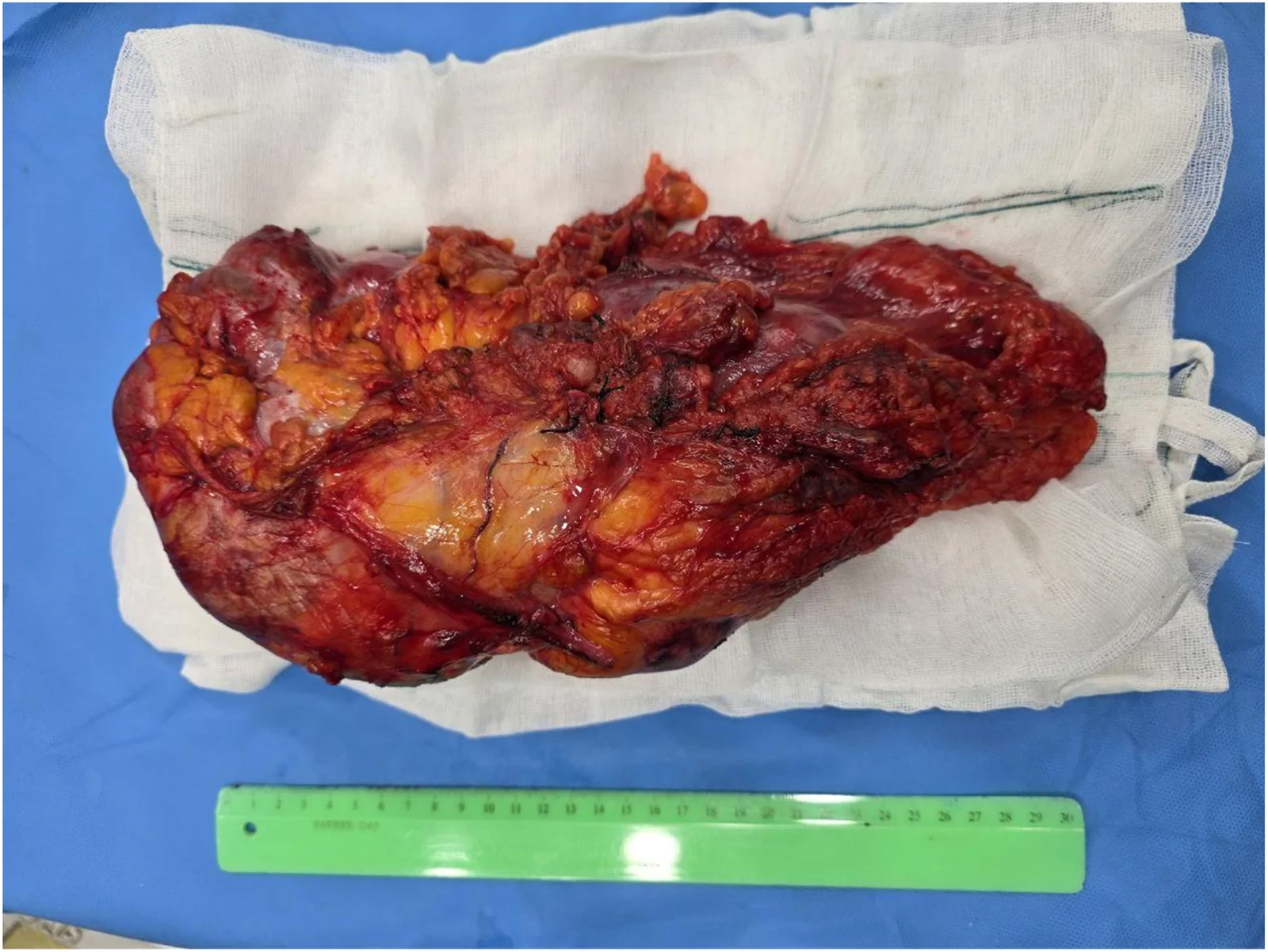
The kidney in the operating room.

The patient underwent nephrectomy. Intraoperatively, dense adhesions between the kidney and the surrounding structures were noted, along with marked inflammation of the renal pedicle. Due to the severity of the inflammatory changes and difficulty in hilar dissection, en bloc ligation of the renal pedicle was performed. Based on the presumptive diagnosis of xanthogranulomatous pyelonephritis (XGP), a simple nephrectomy was completed (Fig. 2).

**Fig. 3:**
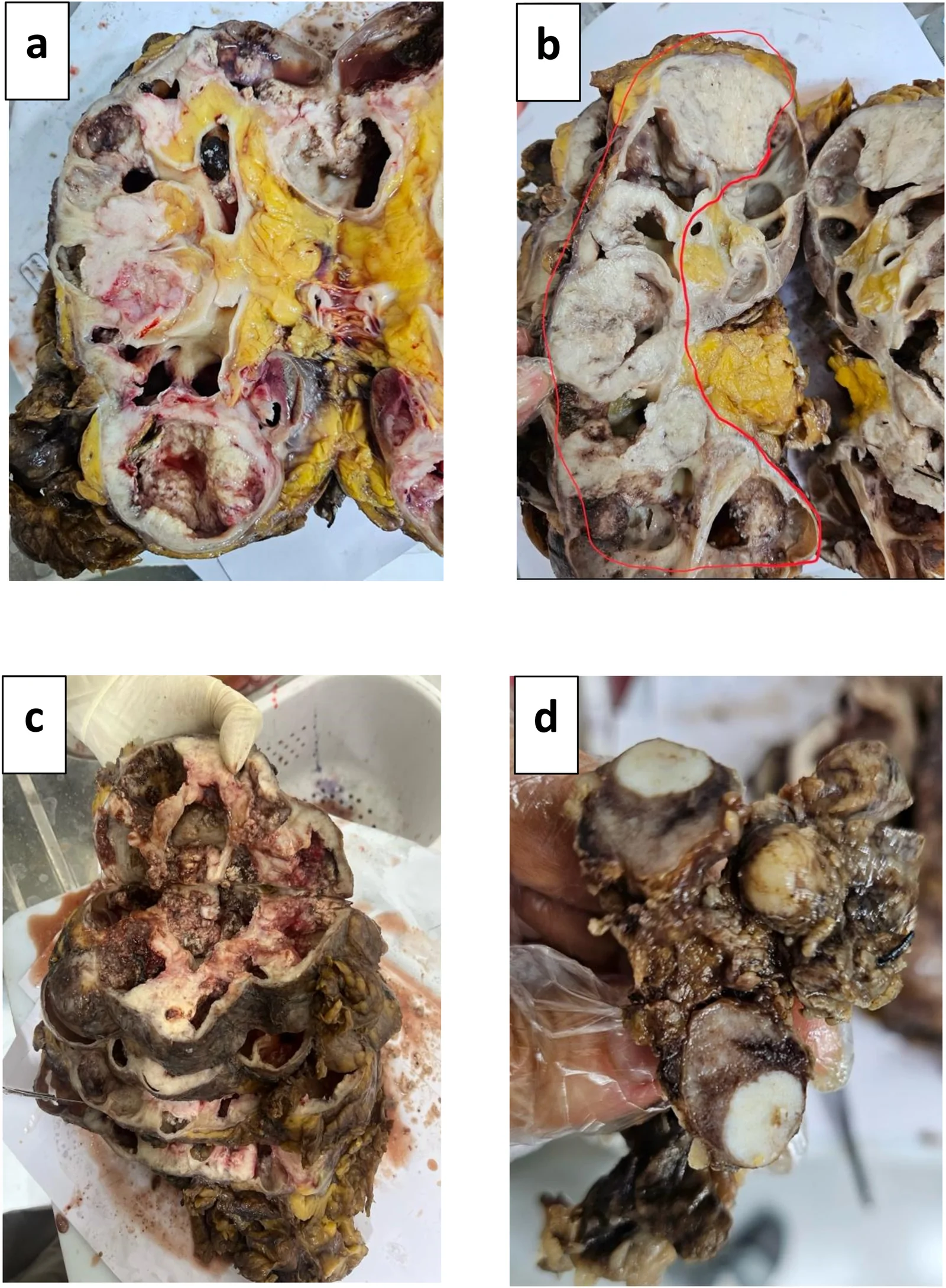
a. Cut surface of the kidney with cyst formation and a blackish stone; b. Tumor borders; c. Wide areas of necrosis; d. Tumor deposit in a hilar lymph node.

The nephrectomy specimen received in the pathology laboratory consisted of a markedly enlarged kidney, measuring 21 × 12 × 10 cm. On sectioning, multiple cystic spaces containing clear tan fluid were identified throughout the kidney. A whitish mass-like lesion replaced the entire renal parenchyma with wide areas of necrosis. Two blackish calculi with abundant sand-like material were present within, along with an extremely dilated pelvicocaliceal system. Several enlarged lymph nodes were identified at the renal hilum, the largest measuring 2.5 cm in greatest dimension. Sectioning and gross examination revealed suspicious areas of tumoral involvement with apparent extension into the renal sinus fat. On dissecting the lymph nodes, one node demonstrated a tumoral deposit measuring 0.8 cm (Fig. 3).

**Fig. 4:**
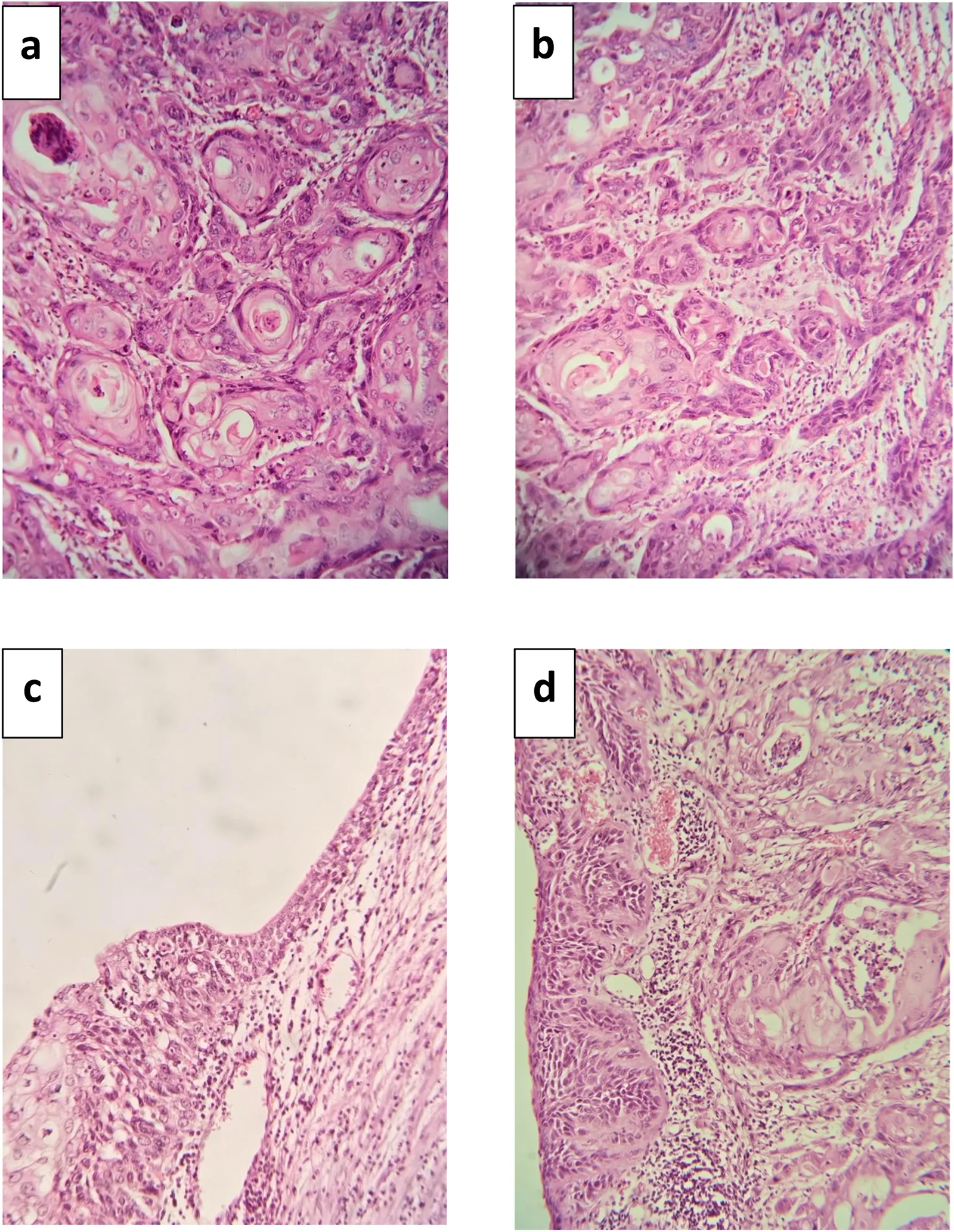
a & b. Well differentiated SCC with abundant keratinization; c. Squamous metaplasia of urothelial lining; d. Carcinoma in situ and invasion.

Microscopic examination revealed a well-differentiated squamous cell carcinoma (SCC) involving the kidney, extending from the upper pole to the lower pole. The tumor exhibited extensive keratinization (Fig. 4), with approximately 30-35% of the tumor showing coagulative necrosis. Prominent areas of squamous metaplasia were identified in the urothelial lining of the cystic spaces. Although the urothelial epithelium demonstrated no evidence of dysplasia, the metaplastic squamous epithelium showed full-thickness dysplasia consistent with carcinoma in situ (CIS) (Fig. 4). Despite extensive sampling, no components of renal cell carcinoma or urothelial (transitional) differentiation were found.

One enlarged lymph node showed a 0.8cm metastatic deposit of tumor without extranodal extension. The tumor involved the renal capsule; however, the renal vessels, ureter, perinephric fat, and attached adrenal gland were free of tumor. The limited residual non-neoplastic kidney showed severe chronic tubulointerstitial inflammation, tubular atrophy, and focal xanthomatous inflammation.

In an immunohistochemical (IHC) study of this case, the markers p63 and CK5/6 were positive in tumor cells, whereas GATA3 was negative; thus, the diagnosis of SCC was confirmed.

Following the histopathological diagnosis of renal SCC, postoperative staging was performed via chest and abdominal CT scans. Chest CT revealed no evidence of pulmonary metastasis. Abdominal CT identified a soft tissue mass within the nephrectomy bed; however, a subsequent core needle biopsy with immunohistochemical (IHC) evaluation confirmed this to be benign and secondary to postoperative changes, with no evidence of malignancy or local recurrence. Although adjuvant chemotherapy was advised by the oncology team, the patient was non-compliant and elected to discontinue both medical treatment and routine post-operative surveillance starting five months ago.

## 3 Discussion

Primary squamous cell carcinoma of the kidney is a rare malignancy, comprising approximately 0.5–8% of all renal cancers and 6–15% of upper urinary tract neoplasms.^3^ That accounts for 0.7 to 7% of all urothelial tumors. A female predominance is reported in some studies,^2^ while others have shown a higher incidence in males.^1^ Chronic irritation of the kidney parenchyma may induce squamous metaplasia of the urothelial lining followed by dysplasia and malignant transformation. The findings were widely observed in the present case.^3^ Long-standing nephrolithiasis, particularly staghorn calculi, is one of the most commonly reported predisposing factors in similar cases.^3^^,^^4^

The clinical manifestations of renal SCC are typically vague and nonspecific, often leading to delayed diagnosis. Patients may present with microscopic or macroscopic hematuria, flank pain, weight loss, fever, or a palpable abdominal mass.^3^

SCC has also been associated with paraneoplastic syndromes, including hypercalcemia, leukocytosis, and thrombocytosis.^3^

Our patient presented with intermittent flank pain, nausea, vomiting, and fever, but did not have gross hematuria or paraneoplastic phenomena.

The radiologic features of primary renal squamous cell carcinoma are largely nonspecific and commonly include hydronephrosis, obstructing renal calculi, and regional lymphadenopathy.^5^^,^^6^ These findings are shared by both inflammatory and neoplastic renal processes.

Given their significantly higher prevalence in the general population, chronic inflammatory conditions such as xanthogranulomatous pyelonephritis (XGP) and renal tuberculosis were initially favored in this patient. Nevertheless, the presence of enhanced renal soft-tissue thickening and multiple enlarged, pathologic lymph nodes raised concern for a superimposed neoplastic process arising in the background of long-standing inflammation.

Histopathologic examination remains the gold standard for diagnosis.^7^ Microscopically, renal SCC exhibits features similar to well-differentiated SCC at other sites, including squamous differentiation, prominent keratinization, and areas of necrosis.^4^

Due to nonspecific clinical and radiologic presentation, this rare entity is often diagnosed at advanced stages, frequently with regional or distant metastasis. In our case, one of the four dissected renal hilar lymph nodes showed a metastatic deposit of 0.8cm of the tumor. Similarly, Takanashi et al. reported hepatic invasion by renal SCC at the time of diagnosis,^8^ and Hsu CJ et al. also described a case of renal pelvis SCC presenting with disseminated metastatic disease.^9^ Consequently, the overall prognosis remains poor.^10^

Although some reports indicate that no standard treatment protocol exists for renal SCC, radical nephrectomy is generally considered the mainstay of treatment when the tumor is confined to the kidney.^1^^,^^11^ In advanced cases, cisplatin-based chemotherapy and palliative radiotherapy have been utilized^1^; however, some studies have not demonstrated a significant survival benefit with these treatment modalities.^12^

Postoperative radiotherapy has generally not shown a survival benefit in patients with upper urinary tract malignancies.^13^ However, some studies have suggested a potential benefit of adjuvant chemotherapy in patients with upper urinary tract urothelial carcinoma.^14^

Overall survival in patients with upper urinary tract SCC is significantly worse compared with those with urothelial carcinoma (UC). However, when matched stage for stage, there is no significant difference in disease-specific five-year survival between SCC and UC.^15^

Surgical intervention at an early stage offers the best chance for curative treatment and improved outcomes.^1^

Due to the rarity of cases and the poor long-term survival associated with renal SCC, further studies are needed to determine whether chemotherapy or radiotherapy improves patient outcomes.

## 4 Conclusion

Squamous cell carcinoma (SCC) in the kidney is extremely rare. It is strongly associated with chronic renal insult secondary to nephrolithiasis and long-standing inflammation, which can promote squamous metaplasia of the urothelial lining, leading to an initiating point for the pathogenesis of SCC. Regarding vague clinical symptoms and significant radiologic overlap with inflammatory renal conditions, this entity is frequently diagnosed at advanced stages. Radical nephrectomy remains the mainstay of treatment. This clinical vignette underscores the importance of maintaining a high index of suspicion for an underlying neoplastic process in patients with chronically irritated non-functional kidneys, and whenever a renal mass is coexistent with renal calculi.^10^

## CRediT authorship contribution statement

**Dorsa Ghasemi:** Writing – review & editing, Writing – original draft, Supervision, Project administration, Methodology. **Milad Benam:** Writing – original draft. **Marjan Akhavan:** Writing – original draft. **Seyed Arman Mousavi:** Writing – original draft. **Arash Sharghi Goorabi:** Writing – original draft. **Soha Ghasemi:** Writing – review & editing.

## Ethics declaration

Written informed consent to take part in the study and to publish the article has been obtained from all participants or their legal representatives. The privacy rights of participants have been observed.

This study included organ or tissue donors. This study includes human biological material and consent was obtained by donors, or their next of kin or legal representatives, for use in this study and for publication of the article. The samples used in this research were not sourced from executed prisoners or prisoners of conscience.

This study was performed in compliance with relevant laws, regulatory frameworks and guidelines where the research took place. Ethics committee approval was not required under relevant laws and institutional guidelines. Our institution does not require ethical approval for reporting individual cases or case series.

This research follows the CARE guidelines and the CARE checklist.

## Ethical considerations

Our institution does not require ethical approval for reporting individual cases or case series.

## Consent for publication

Verbal consent was obtained from the patient, as there is no identifying information.

## Funding

The authors received no financial support for the research, authorship, and/or publication of this article.

## Declaration of conflicting interests

The authors declared no potential conflicts of interest with respect to the research, authorship, and/or publication of this article.
